# Digital Phenotyping in Health Research: Scoping Review of Methods, Gaps, and Opportunities

**DOI:** 10.2196/91747

**Published:** 2026-08-27

**Authors:** Nolwenn Badier, Alice Lafitte, Audrey Difernand, Gloria A Aguayo, Guy Fagherazzi, Jukka-Pekka Onnela, Benjamin Vittrant, Bastien Lechat, Quentin De Larochelambert, Jean-François Toussaint, Lidia Delrieu

**Affiliations:** 1 Institut de Recherche bio-Médicale et d'Epidémiologie du Sport Paris, Île-de-France France; 2 Deep Digital Phenotyping Research Unit, Department of Precision Health Luxembourg Institute of Health Strassen, Luxembourg Luxembourg; 3 Department of Biostatistics Harvard University Boston, MA United States; 4 Withings (France) Issy-les-Moulineaux, Île-de-France France; 5 Sleep Health Flinders Health and Medical Research Institute Flinders University Adelaide, South Australia Australia; 6 Center for Investigations in Medicine and Sports Hôtel-Dieu de Paris Paris, Île-de-France France

**Keywords:** data analysis, digital health, phenotype, review, wearable devices

## Abstract

**Background:**

Wearable and connected digital devices continuously generate large volumes of real-world behavioral, physiological, and environmental data, offering new opportunities for health monitoring and personalized care. Digital phenotyping has emerged as a promising paradigm; yet, there is limited consensus regarding how such data should be analyzed. Inconsistent analytical practices and insufficient methodological reporting may compromise reproducibility, comparability, and the validity of findings.

**Objective:**

This scoping review examined current analytical practices in digital phenotyping in health research, identified methodological gaps, and highlighted the need for transparency and standardized approaches.

**Methods:**

Literature searches were conducted in PubMed, Scopus, Embase, and Web of Science, with Google Scholar used as a complementary source. Eligible studies were published up to December 31, 2024, involved human populations, and used wearable digital devices in longitudinal health research. Studies were screened independently by multiple reviewers using predefined eligibility criteria. Data extraction focused on 6 methodological domains: sample size determination, variable selection and definition, data cleaning and preprocessing, digital phenotyping methods, predictive modeling, and statistical significance handling in large-scale data contexts.

**Results:**

A total of 162 studies were included. Most studies were published from 2018 onward (n=144, 89%) and were conducted primarily in North America (n=92, 57%). Activity trackers (n=81, 50%), smartphones (n=35, 22%), accelerometers (n=26, 16%), and smartwatches (n=24, 15%) were the most frequently used devices. The most common outcomes were activity level (n=67, 41%), sleep (n=65, 40%), step counts (n=63, 39%), and heart rate (n=46, 28%). Heterogeneity and limited reporting were observed across all methodological domains. Preprocessing was the most frequently reported component (n=88, 54%), although specific aspects such as missing-data handling remained inconsistently described (n=35, 22%). Sample size determination methods were reported in only 30% (n=48) of studies, and variable selection methods were described in 27% (n=43). Digital phenotyping approaches were identified in 30% (n=48) of studies and predominantly used regression-based models. Predictive modeling approaches were reported in 17% (n=28) of studies, with substantial diversity in algorithms and limited reporting of validation procedures. Only 4.3% (n=7) of studies explicitly discussed statistical challenges related to large or high-dimensional datasets. Practices varied across health fields, with no domain consistently demonstrating comprehensive reporting across all methodological components.

**Conclusions:**

Digital phenotyping research is expanding rapidly, but methodological practices remain heterogeneous and insufficiently standardized. By providing a cross-domain overview of how wearable-derived longitudinal data are currently processed and analyzed in health research, this review highlights recurring gaps in the reporting and justification of analytical choices, particularly regarding sample size determination, preprocessing, variable definition, predictive modeling, and statistical inference. These findings emphasize the need for clearer analytical frameworks and more consistent reporting practices to improve transparency, reproducibility, and methodological rigor in wearable-based digital health research.

**Trial Registration:**

PROSPERO CRD420251234000; https://www.crd.york.ac.uk/PROSPERO/view/CRD420251234000

## Introduction

### Rationale

Digital devices, such as smartphones, smartwatches, fitness trackers, and other wearable devices, have become deeply integrated into everyday life. Beyond convenience, they continuously generate vast streams of social, behavioral, and physiological data in real-world environments. This data provides unique opportunities to gain deeper insights into human behavior and health, with the potential to transform diagnosis, monitoring, and treatment strategies [1].

Digital phenotyping was initially defined as the “moment-by-moment quantification of individual-level human behavior and physiology using data collected from personal digital devices” [2,3]. Since then, the concept has expanded beyond various active and passive data streams to also include digital assessments, such as digital cognitive tests [1,4]. Initially applied in psychiatry, digital phenotyping is now being explored across chronic disease management, general health monitoring, and public health research [5,6]. Unlike traditional approaches that rely on population averages, digital phenotyping focuses on individual changes over time, offering a more personalized perspective. Because most data are collected passively in real-world contexts, participant burden is minimized while ecological validity is preserved. These technologies thus enable large-scale, long-term monitoring, making them valuable tools for both personalized medicine and population health research [1,2].

Wearable devices have significantly advanced health research by enabling continuous data collection outside clinical settings [1]. Their affordability and ease of use make them particularly attractive for large-scale studies and research in resource-limited environments [7,8]. By providing longitudinal, real-life valid datasets, they advance both precision medicine and public health by fostering the identification of new phenotypes and the development of predictive algorithms for prevention and intervention [2,7,9,10].

Despite their potential, these technologies present significant challenges. Large, complex, and high-frequency datasets require innovative statistical methods to ensure valid interpretation. Missing data, for instance, can bias results if handled improperly, and simplistic methods such as linear interpolation may produce misleading conclusions [11]. Decisions about variable selection, preprocessing pipelines, and feature engineering are critical steps that can determine the quality and reproducibility of findings [1]. Recent studies have further emphasized the growing complexity of analytical pipelines and the lack of standardization in handling these data [12].

Conventional statistical approaches are also strained in this context. Issues such as determining appropriate sample sizes, addressing variability between individuals, and rethinking the role of statistical significance in big-data settings highlight the need for methodological innovation. Ethical considerations, including privacy and informed consent, must further guide the collection and use of personal digital data. As digital phenotyping continues to expand rapidly across disciplines, the need for structured methodological guidance has become increasingly evident [13]. Ultimately, the true promise of digital devices lies not in the scale of data collected, but in the ability to analyze and interpret the data rigorously to advance research and to deliver meaningful and clinically relevant health solutions.

Previous reviews have explored digital phenotyping across a range of specific contexts, particularly in focused domains such as mental health [14-16] or chronic diseases [17], or by examining specific types of devices or sensing modalities [12], or specific analytical approaches and processing pipelines [18]. These studies provide valuable insights into the use of digital phenotyping within well-defined applications. More recent work has also highlighted the broader clinical potential of digital phenotyping [13]. However, most reviews remain centered on a single clinical domain or methodological perspective. To our knowledge, few studies have taken a broader approach to systematically map the diversity of methodological practices used in digital phenotyping across health domains, identify cross-cutting methodological challenges, and highlight gaps in current analytical methods.

Despite rapid growth, digital phenotyping research remains relatively nascent, with limited consensus on best practices for data processing, modeling, and interpretation. Standardized guidelines are lacking, and robust frameworks for analysis are still under development. This fragmentation is further reinforced by the diversity of study designs, data sources, and analytical strategies used across the field [18]. Consolidating knowledge across methodological domains has therefore become increasingly important in order to guide future research and to ensure that digital phenotyping contributes meaningfully to precision medicine [10].

### Objectives

This scoping review aims to fill these gaps by exploring how digital phenotyping is currently applied in health research, describing the analytical methods used, and highlighting their limitations. This work aims to contribute to the larger effort of harnessing the full potential of digital health data while laying the way for future research to address the challenges that remain.

## Methods

### Overview

Given the novelty, rapid evolution, and methodological diversity of this field, a scoping review was the most appropriate design to capture its current state and future directions [19]. The review followed the methodological framework of Arksey and O’Malley [20] and the Joanna Briggs Institute guidelines [21], and is reported in line with the PRISMA-ScR (Preferred Reporting Items for Systematic Reviews and Meta-Analyses extension for Scoping Reviews) checklist [22] (Multimedia Appendix 1). The search strategy was reported in accordance with the PRISMA-S (Preferred Reporting Items for Systematic Reviews and Meta-Analyses Literature Search Extension) [23] (Multimedia Appendix 2).

### Protocol and Registration

The methods were prespecified in the study protocol registered in the PROSPERO (International Prospective Register of Systematic Reviews) database on January 7, 2026 (CRD420251234000).

### Eligibility Criteria

The eligibility criteria were defined in accordance with the Population-Concept-Context framework recommended by the Joanna Briggs Institute. Studies conducted in human populations were eligible, without restriction on age, sex, or health status. The review focused on the use of wearable digital devices in longitudinal health research, including studies collecting repeated or continuous data over time to investigate health-related outcomes, regardless of the methods used to analyze them. This approach allowed us to examine how such data are used in practice across studies. Studies limited to device or algorithm validation, feasibility, adherence, or protocol descriptions were excluded, as the aim was to capture applied uses of wearable data rather than methodological or technical developments. No restrictions were applied regarding the setting or geographic location, and studies conducted in both clinical and community settings were included. Both observational (eg, cohort) and interventional (eg, randomized controlled trial) study designs were considered. Studies reporting primary or secondary analyses were eligible, provided they met the inclusion criteria. Only peer-reviewed full-text articles published in English were included. Gray literature (eg, reports, theses, conference proceedings, and other non–peer-reviewed material) or unpublished studies were excluded to ensure methodological rigor. To capture the evolution of wearable devices used in health research, no start date restriction was applied, and all records published up to December 31, 2024, were considered.

### Information Sources and Literature Search

The literature search was initially conducted between September 1, 2024, and January 31, 2025, using PubMed (National Library of Medicine). Google Scholar was also used as a complementary source to identify additional potentially relevant studies. Due to the large number of results and limited export functionality of Google Scholar, only the first 200 results sorted by relevance were screened. During the revision process, the search strategy was clarified, harmonized, and extended to additional databases to ensure comprehensive and consistent coverage of the literature. An updated search was conducted between March 24 and May 4, 2026, in PubMed (National Library of Medicine), Scopus and Embase (Elsevier), and Web of Science (Clarivate), using a consistent search framework across all databases.

The search strategy combined controlled vocabulary and free-text keywords related to wearable digital devices (eg, “Wearable Electronic Devices,” “Wearable Devices,” “Mobile Applications,” and “Fitness Trackers”), health (eg, “Health,” “Public Health,” and “Chronic Disease”), and data analysis and methodological approaches (eg, “Data Analysis,” “Statistical Power,” “Processing,” “Imputation,” and “Predictive Models”).

The PubMed search strategy was developed using both MeSH and free-text terms, structured across multiple components to comprehensively capture the different methodological dimensions of interest. To account for the methodological diversity of the field, multiple complementary search components were developed, each targeting specific methodological aspects. This conceptual framework was then consistently adapted for the other databases by adjusting syntax and indexing terms while preserving the same combination of concepts and level of detail. In Scopus, Embase, and Web of Science, comprehensive search strings were constructed using free-text terms applied to title and abstract fields, allowing all components of the search strategy to be integrated into a single combined query per database. This approach ensured that the scope and level of detail of the search were consistent across databases. The search strings used are presented in Multimedia Appendix 3.

Date limits were applied at the search stage to include only studies published up to December 31, 2024. Other eligibility criteria (eg, language and publication type) were applied during the screening process. The search strategy was developed by the authors, was not based on a previously published strategy, and was not peer-reviewed. No study registries were searched. No additional studies were identified through expert consultation. Relevant reviews identified through the search were screened to detect additional eligible studies, and both backward (reference list) and forward (citation tracking) snowballing was applied to ensure comprehensive coverage.

### Selection of Sources of Evidence

All identified articles were imported into the reference management system Zotero [24] and transferred to CADIMA [25], a platform designed to support systematic and scoping reviews [26]. After duplicate removal, a two-step screening process was performed in accordance with PRISMA-ScR guidelines. In the first step, titles and abstracts were screened against predefined eligibility criteria, which had been refined through an initial pilot test. In the second step, full texts of potentially eligible studies were reviewed, with reasons for exclusion recorded.

Three reviewers independently assessed each record (NB, AL, and AD), and disagreements were resolved through discussion or, when necessary, adjudication by a fourth reviewer (LD).

### Data Charting and Data Items

A data-charting form was jointly developed by two reviewers (LD and NB) to determine which variables to extract. Based on the needs identified during their respective research and the lack of available methodological guidance, the form was designed to capture key information relevant to the use of wearable digital devices in health research and to map the methodological approaches used in this field.

A pilot test was conducted on a subset of studies to refine the extraction fields and procedures before applying them consistently across all included studies. The data-charting process was iterative, allowing adjustments to the extraction form as needed during the review. Data extraction was performed by 1 reviewer (NB) using the CADIMA platform, and the extracted data were subsequently verified and approved by a second reviewer (LD) to ensure accuracy and consistency. The resulting extraction table was exported to Microsoft Excel for subsequent analyses.

Extracted variables included (1) bibliographic information (title, authors, and year of publication), (2) study objective (the primary objective when multiple objectives were reported), (3) country where the study was conducted, (4) study population (eg, sample size and key characteristics such as sex- or age-specific populations, when applicable), (5) study duration, (6) type of wearable digital device used, (7) health research field, and (8) relevant methodological approaches. These included details related to six domains: (1) sample size determination (eg, reported parameters such as alpha level, statistical power, or assumptions used for calculation), (2) variable selection and definition (eg, type and number of variables or features considered for subsequent analyses), (3) data cleaning, preprocessing, and transformation (eg, handling of missing data and outliers, normalization, and feature engineering), (4) digital phenotyping methods (eg, handling of longitudinal data to construct behavioral or digital biomarkers or to characterize individual-level patterns over time), (5) predictive modeling (eg, type of models or algorithms used), and (6) significance testing in big-data context (eg, use of *P* values, correction methods, or thresholds for statistical significance). Each methodological domain was considered independently from the others. Their presence or absence was recorded for all included studies, allowing the identification of gaps in reporting and analytical practices.

### Synthesis of Results

A descriptive analysis was conducted to summarize the characteristics of the included studies. Variables examined included publication year, country of origin (based on study population or, if not specified, corresponding author), study population, duration of follow-up, type of wearable device, outcomes assessed, disease focus, and reported methodological approaches.

Quantitative variables were summarized using descriptive statistics (minimum, maximum, mean, SD, median, and IQR), and qualitative variables were reported as counts and frequencies. A gap map was constructed to summarize the reporting of key methodological domains and their associated subdomains. Each item was categorized as “well reported,” “partially reported,” or “rarely reported” based on the proportion of studies reporting each item (>50%, 25%-50%, and <25%, respectively).

Analyses were performed in R software (version 4.5.0, R Foundation for Statistical Computing).

## Results

### Selection of Sources of Evidence

**Figure 1:**
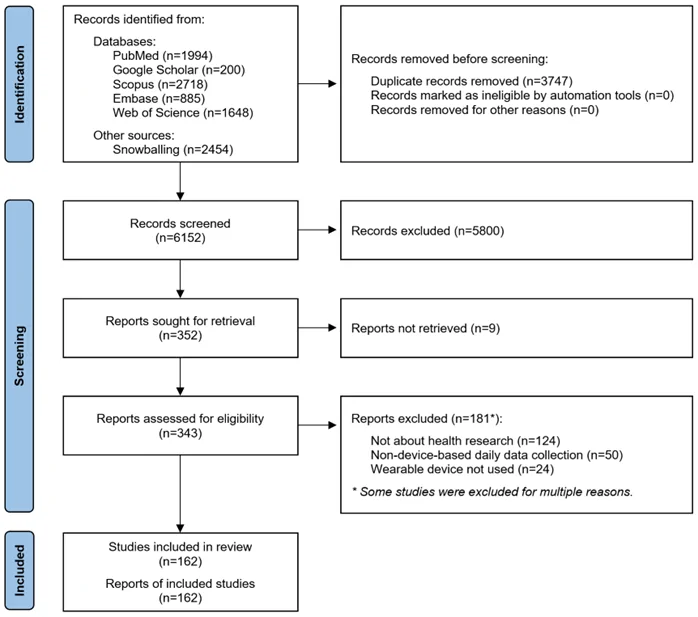
PRISMA (Preferred Reporting Items for Systematic reviews and Meta-Analyses) flow diagram of the study selection process, including identification, screening, eligibility, and inclusion stages.

The search identified 1994 (20%) records from PubMed, 2718 (27%) from Scopus, 885 (8.9%) from Embase, and 1648 (17%) from Web of Science. In addition, the first 200 results from Google Scholar were screened as a complementary source, and 2454 records were identified through snowballing based on 52 relevant reviews, resulting in a total of 9899 records screened. After removing 3747 (38%) duplicates, 6152 abstracts were screened, leading to the exclusion of 5800 (94%) studies. The remaining 352 records were sought for retrieval, of which 9 were not retrieved. A total of 343 full-text articles were assessed for eligibility, and 181 (53%) were excluded. In total, 162 studies met the inclusion criteria and were included in the analysis. The study selection process is shown in Figure 1, and the main characteristics of the included studies are summarized in the Multimedia Appendix 4 [27-188].

### Characteristics of Sources of Evidence

#### Publication Year

The included studies were published between 2005 and 2024 (Table 1). Most studies (n=144, 89%) were published from 2018 onward. The average number of studies per year increased from 4 between 2014 and 2017, to 18 between 2018 and 2023. Publication output further increased in 2024, with 35 (22%) studies.

**Table 1:** Distribution of included studies (N=162) by year of publication (2005-2024).

Year	Value, n (%)
2005	1 (0.6)
2006	0 (0)
2007	0 (0)
2008	0 (0)
2009	0 (0)
2010	1 (0.6)
2011	0 (0)
2012	0 (0)
2013	0 (0)
2014	2 (1.2)
2015	3 (1.9)
2016	5 (3.1)
2017	6 (3.7)
2018	17 (10)
2019	19 (12)
2020	15 (9.3)
2021	18 (11)
2022	23 (14)
2023	17 (10)
2024	35 (22)

#### Countries

The studies were conducted across 23 countries (Table 2), most commonly in North America (n=92, 57%), followed by Europe (n=36, 22%), Asia (n=28, 17%), and Australia (n=6, 3.7%).

**Table 2:** Geographic distribution of included studies (N=162) by country, based on study population or corresponding author when not specified.

Countries	Value, n (%)
United States	85 (53)
Singapore	10 (6.2)
United Kingdom	10 (6.2)
Canada	7 (4.3)
Australia	6 (3.7)
China	5 (3.1)
Japan	5 (3.1)
Republic of Korea	5 (3.1)
Denmark	4 (2.5)
Finland	4 (2.5)
The Netherlands	3 (1.9)
Austria	2 (1.2)
France	2 (1.2)
Ireland	2 (1.2)
Norway	2 (1.2)
Sweden	2 (1.2)
Taiwan	2 (1.2)
Belgium	1 (0.6)
Czechia	1 (0.6)
Germany	1 (0.6)
India	1 (0.6)
Poland	1 (0.6)
Spain	1 (0.6)

#### Populations

**Figure 2:**
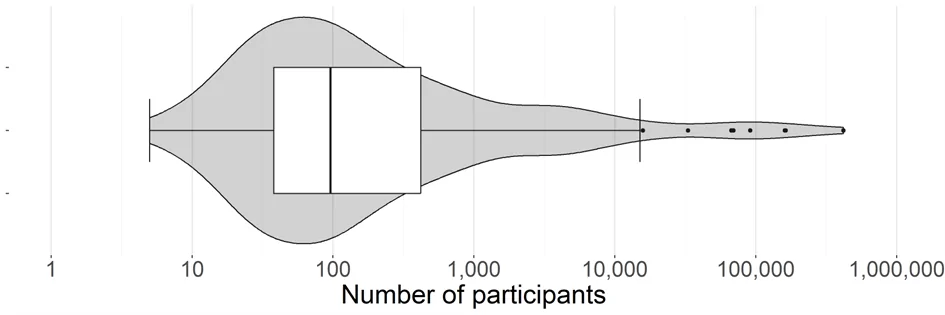
Distribution of sample sizes across included studies (N=162). Sample sizes are displayed on a logarithmic scale to account for variability across studies.

Across the 162 studies, a total of 1,132,068 participants were included, with a median of 96 (IQR 38-420) participants per study (Figure 2). Sample sizes ranged from 5 to 419,093 participants. Most studies enrolled fewer than 100 participants (n=84, 52%), while 49 (30%) included 100-999 participants, 19 (12%) included 1000-9999, 7 (4.3%) included 10,000-99,999, and 3 (1.9%) included more than 100,000 participants.

Most studies (n=99, 61%) included adults aged 18 years and older without targeting a specific subgroup. Others focused on specific populations, including older adults aged 50 years and older (n=20, 12%), workers (n=17, 10%), women (n=10, 6.2%), students (n=10, 6.2%), and children (n=5, 3.1%). Only 1 (0.6%) study specifically focused on men.

#### Devices in Health Research

Most studies used a single wearable device (n=141, 87%). A total of 17 (10%) studies combined 2 devices, and 4 (2.5%) studies included 3 devices.

Across all studies, 11 types of wearable devices were identified. The most common were activity trackers (n=81, 50%), smartphones (n=35, 22%), accelerometers (n=26, 16%), and smartwatches (n=24, 15%). Device categories reflect the terminology used in the original studies. Because reporting standards vary widely, some labels correspond to hardware components (eg, accelerometers), whereas others describe the commercial form factor (eg, smartwatches and activity trackers). As a result, categories may partially overlap. Reported wearing time ranged from 1 day to 16.6 years, with a mean of 39.9 (SD 87.9) weeks.

A total of 10 categories of outcomes were reported. The most frequent were activity level (n=67, 41%), sleep (n=65, 40%), step counts (n=63, 39%), and heart rate (n=46, 28%). Other outcomes included movement patterns (n=14, 8.6%), time of device use (n=8, 4.9%), cardiorespiratory fitness and body composition (n=5, 3.1% each), light exposure (n=2, 1.2%), and blood pressure (n=1, 0.6%).

**Figure 3:**
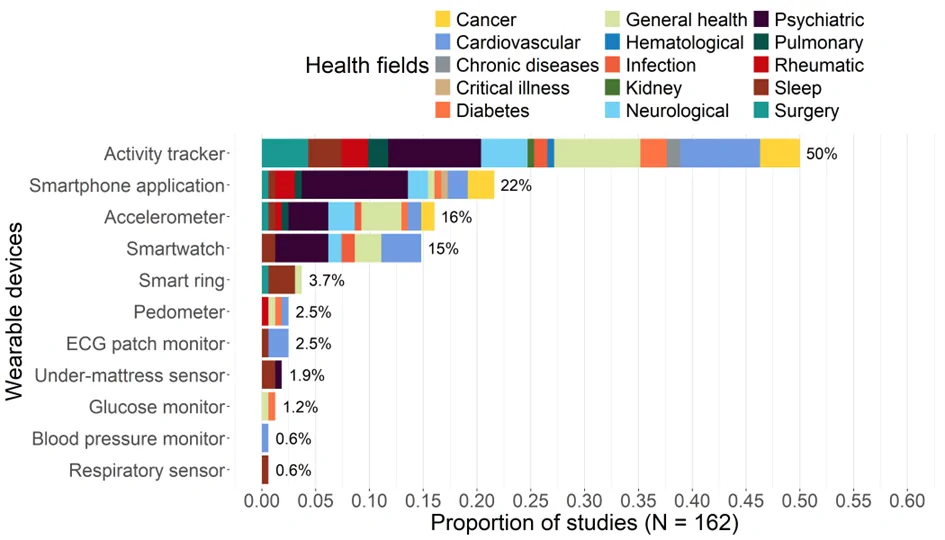
Distribution of wearable device types (n=11) across health research domains (n=15). Bars represent the proportion of studies (N=162) using each type of device, stratified by health field. Multiple device types could be reported within a single study.

Health fields were grouped into 15 categories (Multimedia Appendix 5). The most represented categories were psychiatric disorders (n=41, 25%), general health (n=25, 15%), and cardiovascular diseases (n=23, 14%). Figure 3 shows the distribution of wearable devices across health research domains.

### Results of Individual Sources of Evidence

#### Sample Size Requirements

Of the 162 included studies, 48 (30%) reported a method for determining sample size. Among these, 19 (40%) described the parameters used in the calculation [30,51,55-57,93, 105,108,109,123,149,153,164,166,177,183], while 3 provided justification based on references [62,118,157]. A total of 17 (35%) studies included as many participants as possible [28,40,45,59,63,65,71,76,84,128,132,136,168,170,184,185,188], and 12 (25%) relied on sample sizes reported in previous studies [34,43,44,104,110,111,116,131,140,142,176,180].

Among the 19 studies that specified calculation parameters, expected outcomes were reported in 13 (68%). Among these, effect size was used in 5 (38%), difference rate in 5 (38%), Cohen *d* in 2, and the κ coefficient in 1 study. The type I error rate α was set at .05 in all 14 (74%) studies that reported it. Statistical power was reported in 15 (79%) studies, most often set at 80% (n=12, 80%) or 90% (n=2, 13%), with 1 study using 81%. Attrition was accounted for in 7 (37%) studies, with assumed rates of 10%, 20%, or 25% in 2 (29%) studies each. One study arbitrarily added 8 participants to its final sample size.

Overall, reporting of sample size justification was inconsistent, with variability in the parameters considered and limited reporting of underlying assumptions.

#### Selection and Definition of Variables

Of the 162 included studies, 43 (27%) reported methods for variable selection. Among these, 30 (70%) aggregated longitudinal data into summary measures (eg, means or categories) [41,42,44,45,50,63,75,76,78,87,100,104,109,111, 117,121,137,142,149,154,156,158,159,167,168,171,178,180,182,187], 6 (14%) selected variables based on prior literature [59,81,98,127,170,183], 4 (9.3%) used factorial analysis [92,110,134,164], and 2 (4.7%) applied Pearson correlations [61,138]. One study combined data aggregation with subsequent Pearson correlation analysis [29].

Overall, variable selection approaches were heterogeneous, with a predominant reliance on data aggregation methods, reducing the longitudinal dimension of the data.

#### Data Cleaning, Preprocessing, and Transformations

Of the 162 included studies, 6 (3.7%) cleaned their datasets by removing the first and last day of recording [49,96,109,152,160,165], and 2 others excluded the first 14 days (in 9-month [151] and 2-year studies [183]). Sleep studies applied context-specific rules: 2 studies retained the main sleep episode when multiple episodes were recorded in the same day [84,170], and another excluded specific calendar periods (eg, New Year, Thanksgiving, Christmas, and daylight-saving transitions) [135].

Wear-time validation was reported in 60 (37%) studies. Among these, 38 (63%) required a minimum number of hours per day [32,40,41,44,63,67,73,74,77,81,87,88,90,92,94,96,103,109, 110,112,116,121,122,127,135,138-140,145,150,158,160,167,168,174, 178,180,184], ranging from 1 to 22 hours (mean 9.5, SD 6.0). A total of 32 (53%) studies required a minimum number of valid days [32,43,44,49,54,56,59,62,67,70,77,78,82,87,90,92, 100,110,128,133-135,139,140,142,145,148,165,167,174,183,185], defined based on a prior minimum number of hours, ranging from 1 to 83 days (mean 12.9, SD 19.2). In 3 studies, this requirement depended on study duration (eg, 15 days per month, 24 days during the first 60 days, or 7 days during a 28-day window). In addition, 13 (22%) studies used step counts to confirm wear [28,44,46,48,60,62,67,77,103,128,148,152,162], most often applying a minimum threshold of 100 daily steps (n=8, 62%). Among these, 2 studies required a maximum of 45,000 steps per day, and 2 others a maximum of 50,000 steps per day. Two studies required at least 1000 steps, with 1 also setting a maximum at 20,000 steps per day. Two other studies required at least 1 step per day, and 1 study required 500 steps per day. Two studies based their thresholds on prior literature [81,87], and 2 others used intraclass correlation coefficients ≥0.90 [49,82].

Missing data were addressed in 35 (22%) studies. Among these, 16 (46%) excluded missing data from the analysis [37,41,52,59,78,98,101-104,109,121,125,134,170,181], and 2 (5.7%) applied interpolation methods [143,152], including a 2-value surrounding method. Two other studies replaced missing values with the mean [29,71], and 1 replaced them with the median [132]. In addition, 14 (40%) studies applied imputation methods [31,47,57,61,76,81,86,87,105,119,120,131,155,179], such as multiple imputation (n=4, 12%) or sparse online Gaussian process (n=2, 6%). Five (15%) other studies used regression models, maximum likelihood estimation with the expectation-maximization algorithm, percentile-based methods (97th percentile), k-nearest neighbors, or similar time-of-day segments (1 study each).

A total of 10 (6.2%) studies reported explicit outlier management methods [49,71,96,102,121,132,139,165,180,188]. These included trimming values outside mean ±1 SD (n=3, 30%), z-scores >3 (n=3, 30%), mean ±2 SD (n=1, 10%), and Tukey’s interquartile rule (n=1, 10%). Two studies explicitly retained all data points.

Overall, preprocessing practices were highly heterogeneous, with a wide range of approaches and limited consistency in reporting.

#### Digital Phenotyping Analysis Methods

Of the 162 included studies, 48 (30%) applied digital phenotyping methods. Regression models were the most commonly used methods, reported in 39 (81%) studies [27,36,37,41,46,50,53,61,72,74-77,79,80,84,94,100,102, 104,107,111,117,119,127,137,139,149,156,157,164,165,167, 172,176,179,180,182,185]. Mixed-effects models were the most frequent (n=25, 52%), followed by generalized linear models (n=11, 23%). Other approaches included polynomial models (n=2, 4.2%), as well as generalized estimating equations, survival analysis, cosinor model, and spline-based models (1 study each). Clustering methods were used in 7 (15%) studies [34,65,91,96,134,169,170], including k-means algorithms (n=3, 6.3%), latent class approaches (n=2, 4.2%), and density-based spatial clustering of applications with noise (n=1, 2.1%). One study did not report the clustering algorithm used. Finally, 2 (4.2%) studies applied mixed-effects models to the output of k-means clustering [140,141].

Overall, digital phenotyping analyses relied predominantly on regression-based approaches, with limited use of unsupervised methods.

#### Predictive Models

Of the 162 included studies, 28 (17%) reported the use of predictive modeling approaches, applying a total of 60 models. Most studies (n=16, 57%) relied on a single method [29,40,47,58,69,116,131,139,145,151,154,169,179,181,186,187], while others combined 2 (n=2, 7.1%) [143,170] or 3 (n=6, 21%) methods [43,74,77,78,138,163]. Four additional studies applied 4 [144], 5 [132], 6 [61], and 7 methods [79], respectively.

Among the 28 studies, decision tree-based approaches were the most frequently used, reported in 16 (57%) studies. Random forest was used in 12 (43%) studies, extreme gradient boosting in 11 (39%), gradient boosted trees in 2 (7.1%), and adaptive boosting (n=1, 3.6%). Distance-based methods were reported in 10 studies (36%), including support vector machines (n=8, 29%), k-nearest neighbors (n=6, 21%), and Gaussian processes (n=2, 7.1%). Predictive regression models were used in 4 (14%) studies, including ridge regression, least absolute shrinkage and selection operator, elastic net, and regularized regression (1 study each). Neural network architectures were also reported in 4 (14%) studies and included long-short-term memory and recurrent neural networks (n=2, 7.1% each), as well as gated recurrent units and multilayer perceptrons (1 study each). Sequential models and time-series approaches were each reported in 3 (11%) studies each. All sequential models included Markov chain transitions, while time-series approaches included autoregressive integrated moving average, seasonal autoregressive integrated moving average, and Bayesian structural time series models (1 study each). One study reported the use of an unspecified internal algorithm.

Validation procedures were detailed in 17 (61%) of the 28 studies. A total of 7 (25%) studies used leave-one-subject-out cross-validation [61,80,127,138,143,145,179], and 10 (36%) applied repeated k-fold cross-validation [58,74,77-79,116, 131,139,156,187], with k sometimes specified as 3 (n=1), 4 (n=1), 5 (n=2), or 10 (n=2).

Performance metrics were reported in 10 (36%) studies [58,61,74,77-79,116,138,141,179]. The area under the receiver operating characteristic curve was the most common metric (n=8, 29%). Other metrics included bootstrapped feature stability and *F*_1_ score (n=3, 11% each), confusion matrices, negative predictive value, and κ statistic (n=2, 7.1% each), as well as Shapley values, root-mean-square deviation, Pearson and Spearman correlations, Brier metrics, and mean absolute error (1 study each).

Finally, only 2 (7.1%) studies explicitly discussed model limitations [139,143], particularly the distinction between prediction and causality.

Overall, predictive modeling approaches were diverse but inconsistently reported, with limited transparency regarding validation, performance evaluation, and model limitations.

#### Statistical Significance in Big-Data Contexts

Only 7 (4.3%) studies addressed the limitations of statistical significance testing in a big-data context [50,71,78,108,121,184,185]. Four studies emphasized the need for appropriate adjustment of *P* values, and 1 highlighted that a large number of analyses may increase the risk of spurious findings. Two studies discussed the limitations of their conclusions and stressed the importance of assessing clinical impact beyond statistical significance. They also acknowledged the difficulty of distinguishing relevant from irrelevant correction methods.

Overall, the implications of large-scale data on statistical significance were rarely addressed.

### Methodological Practices Across Health Domains

Methodological practices were further examined across health domains to explore potential differences in reporting patterns (Multimedia Appendix 6). To account for differences in the number of studies across domains, results are presented as proportions within each health domain.

Preprocessing was the most frequently reported methodological component across domains, ranging from 29% (2/7) in surgery-related studies to 64% (9/14) in neurological studies, reaching 60% (3/5) in diabetes and pulmonary-related studies, and 59% (24/41) in psychiatric studies. Sample size planning was inconsistently reported, with proportions ranging from 21% (3/14) in neurological studies to 43% (3/7) in surgical studies, and 40% (2/5) in both diabetes and infection-related studies. It was also reported in 39% (9/23) of cardiovascular studies. Similarly, variable definition was reported in 36% (9/25) of general health and neurological studies (5/14), while lower proportions were observed in cancer (1/10, 10%), surgery (1/7, 14%), and rheumatism (1/6, 17%). Digital phenotyping methods were reported in 44% (11/25) of general health studies, 40% (2/5) in infection-related studies, and 37% (15/41) in psychiatric studies, while remaining below 20% (1/5) in several other domains, including neurological (2/14, 14%) and cancer (1/10, 10%). Predictive modeling was reported in 43% (3/7) of surgical studies and 34% (14/41) of psychiatric studies, but was rare in several domains, including general health (2/25, 8%) and cardiovascular (1/23, 4.3%), and absent in cancer, diabetes, and pulmonary studies. Statistical considerations related to large or high-dimensional data were rarely reported across all domains.

Overall, these results highlight substantial variability in methodological reporting across health domains, with no field consistently demonstrating high levels of reporting across all methodological components.

### Synthesis of Results

The synthesis of methodological practices across included studies highlights several recurring patterns and gaps in the application of digital phenotyping in health research.

Overall, reporting of key methodological components was inconsistent across domains. Sample size justification was rarely described, and when reported, relied on heterogeneous parameters with limited transparency. Similarly, variable selection approaches were highly diverse, with a predominant reliance on data aggregation methods, often reducing the longitudinal dimension of the data. Preprocessing strategies showed substantial variability, with a wide range of approaches for wear-time validation, missing data handling, and outlier management, and no clear consensus on best practices. These inconsistencies were further reflected in analytical choices. While digital phenotyping analyses were predominantly based on regression models, the use of more advanced or unsupervised approaches remained limited. In studies applying predictive modeling, a wide diversity of algorithms was observed, but reporting of validation procedures, performance metrics, and model limitations remained incomplete. In particular, only a small proportion of studies explicitly addressed issues related to overfitting, model generalizability, or the distinction between prediction and causal inference. Finally, the implications of large-scale and high-dimensional data on statistical inference were rarely discussed. Only a few studies addressed challenges related to multiple testing or the interpretation of statistical significance, despite their importance in big-data contexts.

**Figure 4:**
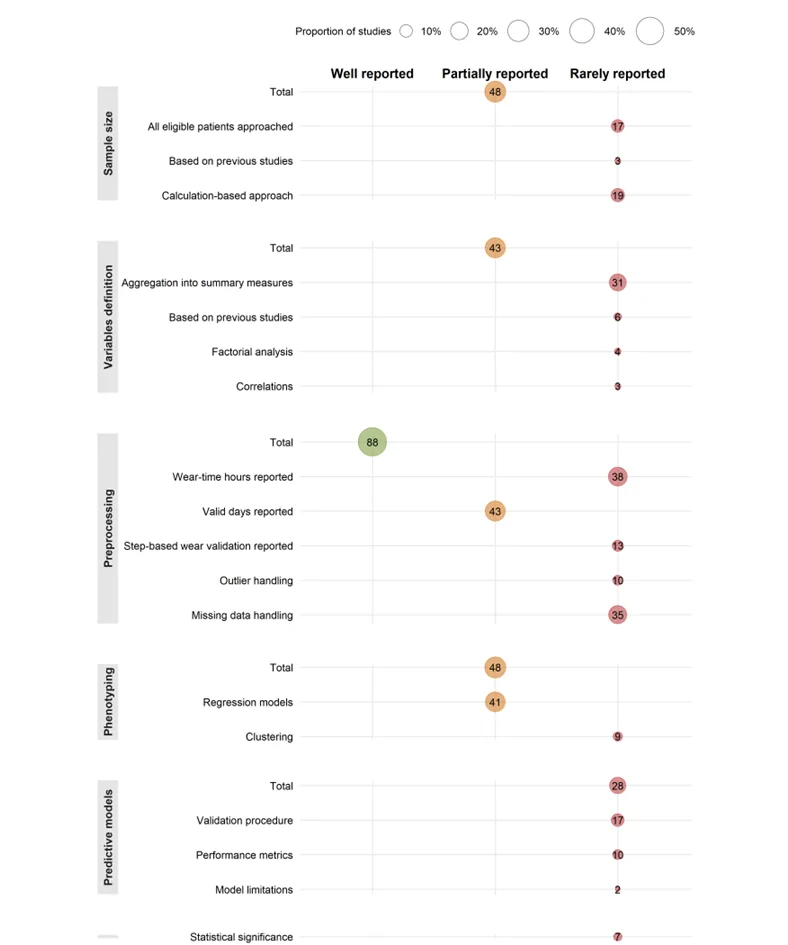
Gap map of methodological reporting across key domains in studies using wearable digital devices in health research (N=162). Reporting levels were categorized as “well reported” (>50% of studies), “partially reported” (25%-50%), and “rarely reported” (<25%). For each domain, the “Total” category indicates whether at least 1 methodological component within the domain was reported in the study.

To provide a structured overview of these methodological gaps, Figure 4 presents a gap map summarizing the extent and consistency of reporting across key methodological domains.

Taken together, these findings highlight a lack of standardization and transparency in methodological practices, as well as important gaps in the reporting and justification of analytical choices. Despite the richness and high frequency of longitudinal data collected through wearable devices, many studies relied on aggregated summary measures, thereby limiting the ability to fully capture temporal dynamics and individual variability. These patterns suggest the need for clearer methodological guidance to support the robust and reproducible use of digital phenotyping in health research.

## Discussion

### Summary of Evidence

This scoping review aimed to examine how digital phenotyping is currently applied in health research, with a focus on analytical methods and their reporting. Overall, the findings reveal substantial heterogeneity and limited transparency across key methodological domains, including sample size determination, variable selection, data preprocessing, and analytical approaches. Despite the increasing availability of rich longitudinal data, many studies relied on simplified or insufficiently described methods, and explicit digital phenotyping approaches were only applied in a minority of studies. These findings highlight important gaps in current methodological practices and emphasize the need for clearer guidance to support robust and reproducible use of wearable-derived data in health research.

Sample size determination was often insufficiently reported and lacked transparency across studies. When described, the underlying assumptions and parameters varied substantially, reflecting the absence of methodological standardization in the field. This heterogeneity may reflect the exploratory nature of many studies, but it also raises important concerns regarding the robustness, reproducibility, and comparability of findings in digital health research [189]. Improving the reporting of sample size justification is therefore essential. Clear documentation of effect sizes, statistical power, significance thresholds, and underlying assumptions is necessary to ensure appropriate study design and interpretation of results [190]. The STROBE (Strengthening the Reporting of Observational Studies in Epidemiology) guidelines provide a useful framework to improve transparency in observational studies and should be more consistently applied in this field [191]. Wider adoption of such frameworks would enhance consistency and support the generation of more robust and generalizable evidence in digital phenotyping research. However, in the context of digital phenotyping, these considerations are further complicated by the longitudinal and high-frequency nature of the data, where sample size requirements also depend on follow-up duration and within-subject variability [192].

Variable selection and definition approaches were highly heterogeneous and often insufficiently described across studies. In many cases, the process used to select or construct variables was not clearly reported, limiting the transparency and interpretability of the analyses [193]. This lack of detail makes it difficult to assess methodological rigor or to compare findings across studies. A notable pattern was the frequent use of data aggregation strategies, where longitudinal data were reduced to summary measures such as means or categories. While these approaches simplify analysis, they may also limit the ability to capture temporal dynamics and within-individual variability, which are central to digital phenotyping, and may lead to misinterpretation of effects [194]. More generally, variable selection and definition should be guided by clearly defined research questions rather than by the constraints of device-derived metrics alone. Improved reporting and more thoughtful use of longitudinal data are therefore essential to fully leverage the richness of wearable-derived datasets.

Data cleaning, preprocessing, and transformation practices were highly heterogeneous across studies, with no clear consensus on how wearable-derived data should be processed. This variability was reflected in key steps such as wear-time validation, inclusion criteria, and analytical procedures, limiting comparability across studies and complicating evidence synthesis [8,195]. A major source of heterogeneity relates to the use of proprietary vs raw data. Many studies relied on device-derived metrics, which are often based on proprietary algorithms that may vary across manufacturers and over time, thereby limiting transparency and reproducibility [195,196]. In contrast, access to raw sensor data enables more transparent preprocessing, facilitates harmonization across devices, and supports data pooling, thereby improving reproducibility [195,197]. Explicit reporting of whether analyses are based on raw signals or device-derived summaries should therefore be encouraged in future digital phenotyping research. Handling of missing data was also inconsistent. While some studies applied advanced imputation methods, many relied on simpler approaches such as deletion, averaging, or interpolation, which may introduce bias or lead to information loss [198,199]. In addition, key aspects of missing data handling were often poorly reported, including the level at which imputation was performed and the type of data concerned. This lack of detail limits the interpretability and reproducibility of analytical strategies. More broadly, few studies discussed the underlying mechanisms of missingness or considered alternative approaches such as sensitivity analyses, despite their importance in longitudinal data analysis [198,199]. Outlier handling was rarely described and lacked standardization when reported. Although classical approaches such as threshold-based methods or interquartile rules were occasionally used, their application remained inconsistent [200,201]. Clear and consistent reporting of preprocessing steps, including missing data and outlier management, is therefore essential to improve the reliability and reproducibility of digital health research.

Despite the growing availability of complex digital health data, digital phenotyping remains inconsistently applied in health research. In this review, most studies relied on regression-based models, which are primarily designed to assess associations rather than to capture dynamic behavioral patterns over time [1]. As a result, these approaches may not fully exploit the richness and temporal complexity of high-frequency data generated by wearable devices [1,202]. There is a gap between the definition of digital phenotyping and its implementation in practice. Digital phenotyping is intended to capture individual-level patterns over time, yet in many studies, it is effectively reduced to the use of standard statistical models applied to repeated measurements. Overall, these findings suggest that the field remains exploratory and heterogeneous. Analytical choices often appear to be driven by feasibility and familiarity rather than by clearly defined objectives. This highlights the need for clearer methodological frameworks and an opportunity to develop approaches better suited to the dynamic nature of digital phenotyping data [1].

Predictive modeling approaches were used across studies, with a wide range of methods including tree-based models, distance-based algorithms, regression-based approaches, and neural networks. This diversity reflects the growing interest in machine learning techniques for analyzing wearable-derived data [203]. However, model development and reporting were often incomplete. Key aspects such as validation procedures and performance metrics were not consistently described, limiting the interpretability and reproducibility of the results [204,205]. In addition, the distinction between prediction and other analytical objectives, such as association or causal inference, was rarely discussed. Only a small number of studies explicitly addressed model limitations, including the interpretation of predictive outputs. This lack of clarification raises concerns about how these models are understood and used in practice [206]. Overall, these observations suggest that the main challenge is not the lack of available methods, but how they are applied and reported. Recent guidelines provide a useful framework for the development and validation of prediction models [207], but clearer reporting standards and more consistent application are essential to ensure that predictive models are appropriately developed, validated, and interpreted in digital health research.

Only a small number of studies addressed the limitations of statistical significance testing in a big-data context. When discussed, concerns mainly related to the increased risk of false-positive findings due to multiple testing and the difficulty of applying appropriate correction methods when the number of comparisons is large or not clearly defined [208]. In this context, classical approaches such as Bonferroni correction may be overly conservative, potentially reducing statistical power and obscuring meaningful effects. Some studies also emphasized that statistical significance does not necessarily translate into clinical relevance, highlighting the importance of considering the practical impact of findings alongside *P* values [209]. Overall, these observations suggest that the implications of large-scale data on statistical inference remain insufficiently explored.

Taken together, these findings highlight substantial variability in methodological practices across health domains, with no domain consistently demonstrating high levels of reporting across all components. This suggests that the observed limitations are not confined to particular clinical fields, but rather reflect broader, systemic challenges in the application of digital phenotyping in health research.

### Limitations

This scoping review has several limitations. Despite efforts to maximize coverage, some relevant studies may have been missed, particularly those published in languages other than English. Data extraction was performed by a single reviewer, which may have introduced bias, although verification steps were implemented to limit this risk. No formal quality appraisal or risk-of-bias assessment was conducted, in line with the objectives of scoping reviews, which aim to map the available literature rather than to evaluate study rigor. In addition, the unit of analysis was the individual study rather than the underlying dataset. As a result, multiple publications based on the same data source may have been included. However, this approach is consistent with the scoping nature of the review, which aimed to capture the diversity of methodological practices across studies. Furthermore, this review focused on methodological practices related to data processing and analysis. Other important aspects, such as code and data availability, reproducible pipelines, and benchmark datasets, were not assessed, although they represent key dimensions of standardization and reproducibility in digital phenotyping research. Finally, the substantial heterogeneity in study designs, technologies, and analytical approaches limited the ability to synthesize findings quantitatively or to identify definitive best practices. These limitations should be considered when interpreting the results.

### Conclusions

By exploring methodological practices across health fields, this scoping review provides a broad cross-domain overview of how wearable-derived longitudinal data are currently processed and analyzed in digital phenotyping research. By structuring these approaches into key domains and examining their reporting across studies, this review highlights recurring gaps and substantial heterogeneity in current analytical practices. Despite the increasing availability of high-frequency real-world data, many studies relied on simplified or insufficiently reported methods, particularly regarding sample size determination, preprocessing, variable definition, predictive modeling, and statistical inference. Overall, the rapid expansion of digital phenotyping research appears to outpace the methodological standards needed to support robust and reproducible analyses. These findings emphasize the need for clearer analytical frameworks and more consistent reporting practices to improve transparency, reproducibility, and interpretability in digital phenotyping research. Beyond identifying current limitations, this review provides a foundation for future methodological guidance and may help support the development of more robust and standardized approaches for the analysis of wearable-derived data in health research.

## Data Availability

All data generated or analyzed during this study are included in this published article and its supplementary information files.

## Appendix

Multimedia Appendix 1 PRISMA-ScR checklist.

Multimedia Appendix 2 PRISMA-S checklist.

Multimedia Appendix 3 Search strings used for the records identification.

Multimedia Appendix 4 Main characteristics of the 162 included studies.

Multimedia Appendix 5 Distribution of health research topics in studies.

Multimedia Appendix 6 Distribution of methodological practices across health domains.

## Abbreviations

PRISMA-S: Preferred Reporting Items for Systematic Reviews and Meta-Analyses Literature Search Extension
PRISMA-ScR: Preferred Reporting Items for Systematic Reviews and Meta-Analyses extension for Scoping Reviews
PROSPERO: International Prospective Register of Systematic Reviews
STROBE: Strengthening the Reporting of Observational Studies in Epidemiology
