# Supplementary material for: Digital Phenotyping in Health Research: Scoping Review of Methods, Gaps, and Opportunities
Source: J Med Internet Res. 2026 Aug 27;28:e91747. doi: 10.2196/91747 (PMC13563036; doi:10.2196/91747)
Supplement: Supplementary file 1 [file jmir_v28i1e91747_app1.pdf]

PRISMA-ScR checklist

Section	Item	PRISMA-ScR Checklist Item	Location(s) Reported
**Title**			
Title	1	Identify the report as a scoping review.	Page 1, lines 2-3
**Abstract**			
Structured summary	2	Provide a structured summary that includes (as applicable) background, objectives, eligibility criteria, sources of evidence, charting methods, results, and conclusions that relate to the review questions and objectives.	Pages 1-2, lines 29-70
**Introduction**			
Rationale	3	Describe the rationale for the review in the context of what is already known. Explain why the review questions/objectives lend themselves to a scoping review approach.	Pages 2-4, lines 75-132
Objectives	4	Provide an explicit statement of the questions and objectives being addressed with reference to their key elements (e.g., population or participants, concepts and context) or other relevant key elements used to conceptualize the review questions and/or objectives.	Page 4, lines 133-137
**Methods**			
Protocol and registration	5	Indicate whether a review protocol exists; state if and where it can be accessed (e.g., a Web address); and if available, provide registration information, including the registration number.	Page 4, lines 147-149
Eligibility criteria	6	Specify characteristics of the sources of evidence used as eligibility criteria (e.g., years considered, language, and publication status), and provide a rationale.	Page 4, lines 150-168
Information sources	7	Describe all information sources in the search (e.g., databases with dates of coverage and contact with authors to identify additional sources), as well as the date the most recent search was executed.	Pages 4-5, lines 169-202
Search	8	Present the full electronic search strategy for at least 1 database, including any limits used, such that it could be repeated.	Pages 4-5, lines 169-202 + Multimedia Appendix 1
Selection of sources of evidence	9	State the process for selecting sources of evidence (i.e., screening and eligibility) included in the scoping review.	Page 5, lines 203-211
Data charting process	10	Describe the methods of charting data from the included sources of evidence (e.g., calibrated forms or forms that have been tested by the team before their use, and whether data charting was done independently or in duplicate) and any processes for obtaining and confirming data from investigators.	Pages 5-6, lines 212-240

Data items	11	List and define all variables for which data were sought and any assumptions and simplifications made.	Pages 5-6, lines 212-240
Critical appraisal of individual sources of evidence	12	If done, provide a rationale for conducting a critical appraisal of included sources of evidence; describe the methods used and how this information was used in any data synthesis (if appropriate).	NA
Synthesis of results	13	Describe the methods of handling and summarizing the data that were charted.	Page 6, lines 241-253
**Results**			
Selection of sources of evidence	14	Give numbers of sources of evidence screened, assessed for eligibility, and included in the review, with reasons for exclusions at each stage, ideally using a flow diagram.	Page 7, lines 256-269
Characteristics of sources of evidence	15	For each source of evidence, present characteristics for which data were charted and provide the citations.	Pages 7-10, lines 270-319
Critical appraisal within sources of evidence	16	If done, present data on critical appraisal of included sources of evidence (see item 12).	NA
Results of individual sources of evidence	17	For each included source of evidence, present the relevant data that were charted that relate to the review questions and objectives.	Pages 10-14, lines 320-466
Synthesis of results	18	Summarize and/or present the charting results as they relate to the review questions and objectives.	Pages 14-16, lines 467-500
**Discussion**			
Summary of evidence	19	Summarize the main results (including an overview of concepts, themes, and types of evidence available), link to the review questions and objectives, and consider the relevance to key groups.	Pages 16-18, lines 503-606
Limitations	20	Discuss the limitations of the scoping review process.	Page 18, lines 607-623
Conclusions	21	Provide a general interpretation of the results with respect to the review questions and objectives, as well as potential implications and/or next steps.	Pages 18-19, lines 624-635
**Funding**			
Funding	22	Describe sources of funding for the included sources of evidence, as well as sources of funding for the scoping review. Describe the role of the funders of the scoping review.	Page 19, lines 636-638
