# Supplementary material for: Digital Phenotyping in Health Research: Scoping Review of Methods, Gaps, and Opportunities
Source: J Med Internet Res. 2026 Aug 27;28:e91747. doi: 10.2196/91747 (PMC13563036; doi:10.2196/91747)
Supplement: Supplementary file 2 [file jmir_v28i1e91747_app2.pdf]

PRISMA-S checklist

Section	Item	PRISMA-S Checklist Item	Location(s) Reported
**Information sources and methods**			
Database name	1	Name each individual database searched, stating the platform for each.	Page 5, lines 170-178
Multi-database searching	2	If databases were searched simultaneously on a single platform, state the name of the platform, listing all of the databases searched.	Page 5, lines 170-178
Study registries	3	List any study registries searched.	Page 5, lines 198-199
Online resources and browsing	4	Describe any online or print source purposefully searched or browsed (e.g., tables of contents, print conference proceedings, web sites), and how this was done.	Page 4, lines 164-166
Citation searching	5	Indicate whether cited references or citing references were examined, and describe any methods used for locating cited/citing references (e.g., browsing reference lists, using a citation index, setting up email alerts for references citing included studies).	Page 5, lines 179-194
Contacts	6	Indicate whether additional studies or data were sought by contacting authors, experts, manufacturers, or others.	Page 5, line 199
Other methods	7	Describe any additional information sources or search methods used.	Page 5, lines 199-202
**Search strategies**			
Full search strategies	8	Include the search strategies for each database and information source, copied and pasted exactly as run.	Multimedia Appendix 1
Limits and restrictions	9	Specify that no limits were used, or describe any limits or restrictions applied to a search (e.g., date or time period, language, study design) and provide justification for their use.	Page 4, lines 157-168
Search filters	10	Indicate whether published search filters were used (as originally designed or modified), and if so, cite the filter(s) used.	Page 5, lines 195-197
Prior work	11	Indicate when search strategies from other literature reviews were adapted or reused for a substantive part or all of the search, citing the previous review(s).	Page 5, lines 197-198
Updates	12	Report the methods used to update the search(es) (e.g., rerunning searches, email alerts).	Page 5, lines 174-178
Dates of searches	13	For each strategy, provide the date when the last search occurred.	Page 5, lines 170-178
**Peer review**			
Peer review	14	Describe any search peer review process.	Page 5, lines 197-198
**Managing records**			
Total records	15	Document the total number of records identified from each database and other information sources.	Page 7, lines 257-260
Deduplication	16	Describe the processes and any software used to deduplicate records from multiple database searches and other information sources.	Page 5, lines 204-206
