# Supplementary material for: Digital Phenotyping in Health Research: Scoping Review of Methods, Gaps, and Opportunities
Source: J Med Internet Res. 2026 Aug 27;28:e91747. doi: 10.2196/91747 (PMC13563036; doi:10.2196/91747)
Supplement: Supplementary file 3 [file jmir_v28i1e91747_app3.pdf]

## 1. Search strings used for the records identification in PubMed

***Primary exploratory search***

("Digital Health"[All Fields] OR "mHealth"[All Fields] OR "eHealth"[All Fields] OR "Wearable Electronic Devices"[MeSH Terms] OR "Wearable Devices"[All Fields] OR "Mobile Applications"[MeSH Terms] OR "Mobile Health"[All Fields] OR "Fitness Trackers"[All Fields]) AND ("Health"[MeSH Terms] OR "Public Health"[MeSH Terms] OR "Chronic Disease"[MeSH Terms]) AND ("Data Analysis"[All Fields] OR "Big Data"[MeSH Terms] OR "Artificial Intelligence"[MeSH Terms] OR "Health Information Systems"[MeSH Terms] OR "Data Collection"[MeSH Terms])

"Digital Health"[MeSH Terms] OR "Digital Phenotyping"[MeSH Terms]

("Digital Health"[All Fields] OR "mHealth"[All Fields] OR "eHealth"[All Fields] OR "Wearable Electronic Devices"[MeSH Terms] OR "Wearable Devices"[All Fields] OR "Fitness Trackers"[All Fields]) AND ("Health"[MeSH Terms] OR "Public Health"[MeSH Terms] OR "Chronic Disease"[MeSH Terms]) AND ("Data Analysis"[All Fields] OR "Big Data"[MeSH Terms] OR "Artificial Intelligence"[MeSH Terms])

***Methodological aspects: study design and sample size***

(("Digital Health"[All Fields] OR "mHealth"[All Fields] OR "eHealth"[All Fields] OR "Wearable Electronic Devices"[MeSH Terms] OR "Wearable Devices"[All Fields] OR "Mobile Applications"[MeSH Terms] OR "Mobile Health"[All Fields] OR "Fitness Trackers"[All Fields]) AND ("Health"[MeSH Terms] OR "Public Health"[MeSH Terms] OR "Chronic Disease"[MeSH Terms]) AND ("Data Analysis"[All Fields] OR "Big Data"[MeSH Terms] OR "Artificial Intelligence"[MeSH Terms] OR "Health Information Systems"[MeSH Terms] OR "Data Collection"[MeSH Terms])) AND ("Sample Size"[MeSH Terms] OR "Sample Size"[All Fields] OR "Statistical Power"[MeSH Terms] OR "Power Analysis"[All Fields] OR "Study Design"[MeSH Terms])

***Methodological aspects: variable selection and digital phenotyping***

(("Digital Health"[All Fields] OR "mHealth"[All Fields] OR "eHealth"[All Fields] OR "Wearable Electronic Devices"[MeSH Terms] OR "Wearable Devices"[All Fields] OR "Mobile Applications"[MeSH Terms] OR "Mobile Health"[All Fields] OR "Fitness Trackers"[All Fields]) AND ("Health"[MeSH Terms] OR "Public Health"[MeSH Terms] OR "Chronic Disease"[MeSH Terms]) AND ("Data Analysis"[All Fields] OR "Big Data"[MeSH Terms] OR "Artificial Intelligence"[MeSH Terms] OR "Health Information Systems"[MeSH Terms] OR "Data Collection"[MeSH Terms])) AND (("Feature Selection"[All Fields] OR "Variable Selection"[All Fields] OR "Predictor Variables"[All Fields]) OR ("Digital Phenotyping"[All Fields] OR "Digital Biomarkers"[All Fields] OR "Behavioral Biomarkers"[All Fields]))

***Methodological aspects: data preprocessing***

(("Digital Health"[All Fields] OR "mHealth"[All Fields] OR "eHealth"[All Fields] OR "Wearable Electronic Devices"[MeSH Terms] OR "Wearable Devices"[All Fields] OR "Mobile Applications"[MeSH Terms] OR "Mobile Health"[All Fields] OR "Fitness Trackers"[All Fields]) AND ("Health"[MeSH Terms] OR "Public Health"[MeSH Terms] OR

"Chronic Disease"[MeSH Terms]) AND ("Data Analysis"[All Fields] OR "Big Data"[MeSH Terms] OR "Artificial Intelligence"[MeSH Terms] OR "Health Information Systems"[MeSH Terms] OR "Data Collection"[MeSH Terms])) AND (("Data Cleaning"[All Fields] OR "Data Preprocessing"[All Fields] OR "Data Transformation"[All Fields] OR "Data Preparation"[All Fields]) OR ("Signal Processing"[All Fields] OR "Feature Engineering"[All Fields] OR "Data Imputation"[All Fields]))

***Methodological aspects: predictive modeling***

(("Digital Health"[All Fields] OR "mHealth"[All Fields] OR "eHealth"[All Fields] OR "Wearable Electronic Devices"[MeSH Terms] OR "Wearable Devices"[All Fields] OR "Mobile Applications"[MeSH Terms] OR "Mobile Health"[All Fields] OR "Fitness Trackers"[All Fields]) AND ("Health"[MeSH Terms] OR "Public Health"[MeSH Terms] OR "Chronic Disease"[MeSH Terms]) AND ("Data Analysis"[All Fields] OR "Big Data"[MeSH Terms] OR "Artificial Intelligence"[MeSH Terms] OR "Health Information Systems"[MeSH Terms] OR "Data Collection"[MeSH Terms])) AND (("Predictive Models"[All Fields] OR "Machine Learning"[MeSH Terms] OR "Artificial Intelligence"[MeSH Terms] OR "Prediction Models"[All Fields]) OR ("Digital Phenotyping"[All Fields] OR "Digital Biomarkers"[All Fields] OR "Behavioral Biomarkers"[All Fields]))

***Methodological aspects: statistical significance in large datasets***

(("Digital Health"[All Fields] OR "mHealth"[All Fields] OR "eHealth"[All Fields] OR "Wearable Electronic Devices"[MeSH Terms] OR "Wearable Devices"[All Fields] OR "Mobile Applications"[MeSH Terms] OR "Mobile Health"[All Fields] OR "Fitness Trackers"[All Fields]) AND ("Health"[MeSH Terms] OR "Public Health"[MeSH Terms] OR "Chronic Disease"[MeSH Terms]) AND ("Data Analysis"[All Fields] OR "Big Data"[MeSH Terms] OR "Artificial Intelligence"[MeSH Terms] OR "Health Information Systems"[MeSH Terms] OR "Data Collection"[MeSH Terms])) AND (("p-value"[All Fields] OR "p-values"[All Fields] OR "Statistical Significance"[MeSH Terms] OR "Significance Testing"[All Fields]) AND ("Big Data"[All Fields] OR "Large Datasets"[All Fields] OR "High-Dimensional Data"[All Fields]))

***Combined methodological search***

(("Digital Health"[All Fields] OR "mHealth"[All Fields] OR "eHealth"[All Fields] OR "Wearable Electronic Devices"[MeSH Terms] OR "Wearable Devices"[All Fields] OR "Mobile Applications"[MeSH Terms] OR "Mobile Health"[All Fields] OR "Fitness Trackers"[All Fields]) AND ("Health"[MeSH Terms] OR "Public Health"[MeSH Terms] OR "Chronic Disease"[MeSH Terms]) AND ("Data Analysis"[All Fields] OR "Big Data"[MeSH Terms] OR "Artificial Intelligence"[MeSH Terms] OR "Health Information Systems"[MeSH Terms] OR "Data Collection"[MeSH Terms])) AND (("Sample Size"[MeSH Terms] OR "Statistical Power"[MeSH Terms] OR "Study Design"[MeSH Terms] OR "Power Analysis"[All Fields]) OR ("Feature Selection"[All Fields] OR "Variable Selection"[All Fields] OR "Predictor Variables"[All Fields]) OR ("Data Cleaning"[All Fields] OR "Data Preprocessing"[All Fields] OR "Data Transformation"[All Fields] OR "Signal Processing"[All Fields] OR "Feature Engineering"[All Fields] OR "Data Imputation"[All Fields]) OR ("Predictive Models"[All Fields] OR "Machine Learning"[MeSH Terms] OR "Prediction Models"[All Fields] OR "Digital Phenotyping"[All Fields] OR "Digital Biomarkers"[All Fields] OR "Behavioral Biomarkers"[All Fields]) OR ("p-value"[All Fields] OR "p-values"[All Fields] OR "Statistical Significance"[MeSH Terms] OR "Significance Testing"[All Fields])

AND ("Big Data"[All Fields] OR "Large Datasets"[All Fields] OR "High-Dimensional Data"[All Fields]))

***Additional exploratory search***

("Daily data"[All Fields] AND ("Digital data"[All Fields] OR "Digital Phenotyping"[All Fields] OR "Wearable Electronic Devices"[MeSH Terms])) AND ("Healthcare"[MeSH Terms] OR "Health"[MeSH Terms]) AND ("Methodological Guidelines"[All Fields] OR "Sample Size"[MeSH Terms] OR "Multimodal Approach"[All Fields] OR "Multimodal Analysis"[All Fields] OR "Multimodal Data"[All Fields] OR "High-dimensional Analysis"[All Fields] OR "Missing Data"[MeSH Terms] OR "Z-score"[All Fields] OR "Outliers"[All Fields] OR "Data transformation"[All Fields] OR "Normalization"[All Fields] OR "Machine Learning"[MeSH Terms])

## 2. Search string used for the records identification in Google Scholar

("digital phenotyping" OR "wearable devices" OR "mobile health" OR "mhealth") AND ("longitudinal" OR "daily data" OR "time series")

## 3. Search string used for the records identification in Scopus

TITLE-ABS-KEY(("digital health" OR "mhealth" OR "ehealth" OR "mobile health" OR "wearable devices" OR "wearable electronic devices" OR "fitness trackers" OR "mobile applications" OR "digital phenotyping") AND ("health" OR "public health" OR "chronic disease") AND ("data analysis" OR "big data" OR "artificial intelligence" OR "machine learning" OR "health information systems" OR "data collection") AND ("sample size" OR "statistical power" OR "study design" OR "power analysis" OR "feature selection" OR "variable selection" OR "predictor variables" OR "data preprocessing" OR "data cleaning" OR "data transformation" OR "data preparation" OR "signal processing" OR "feature engineering" OR "data imputation" OR "predictive models" OR "prediction models" OR "machine learning" OR "digital biomarkers" OR "behavioral biomarkers" OR "statistical significance" OR "p-value" OR "p-values" OR "significance testing" OR "large datasets" OR "high-dimensional data"))

## 4. Search string used for the record identification in Embase

(('digital health':ti,ab OR mhealth:ti,ab OR ehealth:ti,ab OR 'mobile health':ti,ab OR 'wearable device*':ti,ab OR 'wearable electronic device*':ti,ab OR 'fitness tracker*':ti,ab OR 'mobile application*':ti,ab OR 'digital phenotyping':ti,ab) AND (health:ti,ab OR 'public health':ti,ab OR 'chronic disease':ti,ab) AND ('data analysis':ti,ab OR 'big data':ti,ab OR 'artificial intelligence':ti,ab OR 'machine learning':ti,ab OR 'health information system*':ti,ab OR 'data collection':ti,ab) AND ('sample size':ti,ab OR 'statistical power':ti,ab OR 'study design':ti,ab OR 'power analysis':ti,ab OR 'feature selection':ti,ab OR 'variable selection':ti,ab OR 'predictor variable*':ti,ab OR 'data preprocessing':ti,ab OR 'data cleaning':ti,ab OR 'data transformation':ti,ab OR 'data preparation':ti,ab OR 'signal processing':ti,ab OR 'feature engineering':ti,ab OR 'data imputation':ti,ab OR 'predictive model*':ti,ab OR 'prediction model*':ti,ab OR 'machine learning':ti,ab OR 'digital biomarker*':ti,ab OR 'behavioral biomarker*':ti,ab OR 'statistical significance':ti,ab OR 'p-value*':ti,ab OR 'significance testing':ti,ab OR 'large dataset*':ti,ab OR 'high dimensional data':ti,ab))

## 5. Search string used for the record identification in Web of Science

(("digital health" OR mhealth OR ehealth OR "mobile health" OR "wearable device*" OR "wearable electronic device*" OR "fitness tracker*" OR "mobile application*" OR "digital phenotyping") AND (health OR "public health" OR "chronic disease") AND ("data analysis" OR "big data" OR "artificial intelligence" OR "machine learning" OR "health information system*" OR "data collection") AND ("sample size" OR "statistical power" OR "study design" OR "power analysis" OR "feature selection" OR "variable selection" OR "predictor variable*" OR "data preprocessing" OR "data cleaning" OR "data transformation" OR "data preparation" OR "signal processing" OR "feature engineering" OR "data imputation" OR "predictive model*" OR "prediction model*" OR "machine learning" OR "digital biomarker*" OR "behavioral biomarker*" OR "statistical significance" OR "p-value*" OR "significance testing" OR "large dataset*" OR "high dimensional data"))
