# Supplementary material for: Digital Phenotyping in Health Research: Scoping Review of Methods, Gaps, and Opportunities
Source: J Med Internet Res. 2026 Aug 27;28:e91747. doi: 10.2196/91747 (PMC13563036; doi:10.2196/91747)
Supplement: Supplementary file 4 [file jmir_v28i1e91747_app5.pdf]

Distribution of health research topics in studies (N = 162)

Health categories	Specific diseases	Number of studies (%)
Psychiatric disorders		41 (25)
	Depression	15 (37)
	Mood disorders	4 (9.8)
	Stress	4 (9.8)
	Unspecified	*4 (9.8)*
	Bipolar disorders	3 (7.3)
	Schizophrenia	3 (7.3)
	Anxiety	2 (4.9)
	Loneliness	2 (4.9)
	Suicidal behavior	2 (4.9)
	Happiness	1 (2.8)
	Panic disorders	1 (2.8)
General health		25 (15)
	Unspecified	*12 (48)*
	Sedentary	2 (8.0)
	Fatigue	1 (4.0)
	Low muscle mass	1 (4.0)
	Menstrual cycle	1 (4.0)
	Mortality	1 (4.0)
	Myopia	1 (4.0)
	Overweight/obesity	1 (4.0)
	Physical activity	1 (4.0)
	Pregnancy	1 (4.0)
	Rehabilitation	1 (4.0)
	Small for gestational age	1 (4.0)
	Smoking	1 (4.0)
Cardiovascular diseases		23 (14)
	Unspecified	*6 (26)*
	Atrial fibrillation	4 (17)
	Heart failure	3 (13)
	Heart rate	3 (13)
	Acute myocardial infarction	1 (4.3)
	Blood pressure	1 (4.3)
	Coronary artery disease	1 (4.3)
	Hypertension	1 (4.3)
	Peripheral artery disease	1 (4.3)
	Pre-frailty risk	1 (4.3)
	Systolic blood pressure	1 (4.3)
Sleep disturbances		16 (9.9)
	Unspecified	*12 (75)*
	Sleep disorders	2 (12.5)
	Insomnia	1 (6.3)
	Obstructive sleep apnea	1 (6.3)
Neurological disorders		14 (8.6)
	Amyotrophic lateral sclerosis	3 (21)
	Cognitive development	2 (14)
	Falls	2 (14)
	Parkinson	2 (14)
	Amnestic mild cognitive impairment	1 (7.1)
	Cognitive development	1 (7.1)
	Covert encephalopathy	1 (7.1)
	Dementia	1 (7.1)
	Multiple sclerosis	1 (7.1)
	Tremor	1 (7.1)

**Health categories**	**Specific diseases**	**Number of studies (%)**
Cancer		10 (6.2)
	Unspecified	*4 (40)*
	Breast	2 (20)
	Brain	1 (10)
	Glioblastoma	1 (10)
	Lung	1 (10)
	Peritoneal carcinomatosis	1 (10)
Surgery recovery		7 (4.3)
	Lumbar spine	3 (43)
	Hip/knee arthroplasty	2 (29)
	Colorectal	1 (14)
	Hip/knee joint replacement	1 (14)
Rheumatic disorders		6 (3.7)
	Osteoarthritis	3 (50)
	Chronic inflammatory	1 (17)
	Osteoporosis	1 (17)
	Spine disease	1 (17)
Diabetes		5 (3.1)
	Type II	3 (60)
	Glucose response	1 (20)
	Type I	1 (20)
Infection		5 (3.1)
	Covid-19	3 (60)
	HIV	1 (20)
	Influenza	1 (20)
Pulmonary diseases		5 (3.1)
	Chronic obstructive pulmonary disease	3 (60)
	Arterial hypertension	1 (20)
	Asthma	1 (20)
Chronic diseases		2 (1.2)
	Pancreatitis	1 (50)
	Unspecified	*1 (50)*
Critical illness		1 (0.6)
	Burn	1 (100)
Hematologic disease		1 (0.6)
	Paroxysmal nocturnal hemoglobinuria	1 (100)
Kidney		1 (0.6)
	Hemodialysis	1 (100)
