# Supplementary material for: Digital Phenotyping in Health Research: Scoping Review of Methods, Gaps, and Opportunities
Source: J Med Internet Res. 2026 Aug 27;28:e91747. doi: 10.2196/91747 (PMC13563036; doi:10.2196/91747)
Supplement: Supplementary file 5 [file jmir_v28i1e91747_app6.pdf]

Distribution of methodological practices across health domains (N = 162).

Percentages represent the proportion of studies within each health domain reporting each methodological component.

Health fields	Studies (N = 162)[1]	Sample size planning (N = 48)[1]	Variables definition (N = 43)[1]	Preprocessing (N = 88)[1]	Digital phenotyping methods (N = 48)[1]	Predictive modeling (N = 28)[1]	Statistical significance (N = 7)[1]
**Psychiatric**	41 (25)	10 (24)	8 (20)	24 (59)	15 (37)	14 (34)	1 (2.4)
**General health**	25 (15)	6 (24)	9 (36)	13 (52)	11 (44)	2 (8.0)	1 (4.0)
**Cardiovascular**	23 (14)	9 (39)	6 (26)	12 (52)	5 (22)	1 (4.3)	1 (4.3)
**Sleep**	16 (9.9)	4 (25)	5 (31)	9 (56)	4 (25)	3 (19)	1 (6.3)
**Neurological**	14 (8.6)	3 (21)	5 (36)	9 (64)	2 (14)	2 (14)	0 (0.0)
**Cancer**	10 (6.2)	3 (30)	1 (10)	5 (50)	1 (10)	0 (0.0)	1 (10)
**Surgery**	7 (4.3)	3 (43)	1 (14)	2 (29)	2 (29)	3 (43)	0 (0.0)
**Rheumatic**	6 (3.7)	2 (33)	1 (17)	3 (50)	2 (33)	1 (17)	0 (0.0)
**Diabetes**	5 (3.1)	2 (40)	1 (20)	3 (60)	1 (20)	0 (0.0)	0 (0.0)
**Infection**	5 (3.1)	2 (40)	2 (40)	2 (40)	2 (40)	1 (20)	0 (0.0)
**Pulmonary**	5 (3.1)	1 (20)	2 (40)	3 (60)	1 (20)	0 (0.0)	0 (0.0)
**Chronic diseases**	2 (1.2)	2 (100)	0 (0.0)	2 (100)	1 (50)	1 (50)	1 (50)
**Critical illness**	1 (0.6)	0 (0.0)	1 (100)	0 (0)	1 (100)	0 (0.0)	1 (100)
**Hematological**	1 (0.6)	1 (100)	1 (100)	1 (100)	0 (0.0)	0 (0.0)	0 (0.0)
**Kidney**	1 (0.6)	0 (0.0)	0 (0.0)	0 (0)	0 (0.0)	0 (0.0)	0 (0.0)

[1] n (%)
